# Feeding an Essential Oil Blend to Growing Crossbred Lambs Mitigates Heat Stress to Improve Growth Performance via Enhanced Antioxidant Capacity

**DOI:** 10.3390/ani16050853

**Published:** 2026-03-09

**Authors:** Yannan Ma, Lei Yang, Fan Wu, Jiao Luo, Zhixian Liu, Wen Chen, Zhaomin Lei, Pengjia He, Ting Liu, Shuzhen Song, Shuai Wang, Jianping Wu, David P. Casper

**Affiliations:** 1 College of Life Sciences, Northwest Normal University, Lanzhou 730070, China; mayannan@nwnu.edu.cn (Y.M.); 2023222833@nwnu.edu.cn (L.Y.); 2023222822@nwnu.edu.cn (J.L.); liuzhixian@nwnu.edu.cn (Z.L.); skychw@nwnu.edu.cn (W.C.); wangshuaiyouxiang1@126.com (S.W.); 2 Institute of New Rural Development, Northwest Normal University, Lanzhou 730070, China; 3 College of Chemistry and Chemical Engineering, Wuhan University of Science and Technology, Wuhan 430081, China; wufan46k@163.com; 4 College of Animal Science and Technology, Gansu Agricultural University, Lanzhou 730070, China; leizm@gsau.edu.cn (Z.L.); hepengjia23@163.com (P.H.); liuting0628@163.com (T.L.); 5 Gansu Academy of Agricultural Sciences, Lanzhou 730070, China; songshuzhen@gsagr.ac.cn; 6 Casper’s Calf Ranch, LLC, 4890 W. Lily Creek Road, Freeport, IL 61032, USA

**Keywords:** essential oil blend, growth performance, heat-stress, lambs, health, immunity

## Abstract

Heat stress (HS) poses a significant stress challenge to growing lambs, impacting growth performance, health, and physiological responses. The study evaluated feeding an essential oil blend (EOB) on growth performance, physiological and blood parameters, oxidative, and immune responses. Thirty-two 3 mo old female (BW 18.6 ± 2.43 kg) crossbred (Mongolian × Thin-tailed Han F1) lambs were randomly assigned to either: (1) control (CON)—grain mix without EOB; or (2) EOB—an EOB blend supplemented at 4 g/kg grain mix. The EOB blend was comprised of 4.34% Zanthoxylum, 1% capsicum, and 1.06% cinnamon oils with 93.6% attapulgite carrier containing linalool, sabinene, limonene, capsaicin, cinnamaldehyde and eugenol. Extreme heat stress occurred for 39 out of the 45 d experiment. Feeding HS lambs EOB increased (*p* < 0.05) ADG (107.4 and 162.0 g/d for CON and EOB, respectively), forage intake (239.2 and 287.0 g/d), DMI (863.1 and 930.2 g/d), and feed efficiency (0.123 and 0.181 ADG, g/DMI, g) compared with lambs fed CON. Feeding EOB reduced (*p* < 0.01) respiration rates (RR) and rectal (RT) and skin temperatures (ST) compared to CON-fed lambs. Lambs fed EOB had a higher T-SOD activity and IgM concentration (0.05 < *p* < 0.10) than CON-fed lambs. Growing heat-stressed lambs fed EOB demonstrated a lower (*p* < 0.05) eosinophil percentage. Feeding EOB ameliorated HS conditions for growing crossbred lambs, which demonstrate improved growth performance, enhanced physiological responses, and overall health status.

## Simple Summary

Heat poses a significant stress challenge to sheep farming around the world, especially in arid and semi-arid regions, impacting lamb growth performance, health, and physiological status. This experiment evaluated feeding an essential oil blend, as a potential feed supplement, to ameliorate HS in thirty-two female growing lambs measuring growth performance, blood metabolites, antioxidant capacity, and physiological responses. Treatments were a control and an essential oil blend supplemented at 4 g/kg in the concentrate mix. Lambs were fed separately free-choice concentrate mix and forage mix. Lambs experienced extreme heat stress for 39 of the 45 d experiment. Growing heat-stressed lambs fed essential oil blend demonstrated approximately 50% improvement in body weight gain compared with control fed lambs. Forage, dry matter intake, and feed efficiency were greater for essential oil blend fed lambs compared with control fed lambs. Respiration rates, rectal temperatures, and skin temperatures were lower for lambs fed the essential oil blend compared with control fed lambs. The feeding of an essential oil blend as a dietary feed supplement at 4 g/kg concentrate mix has the potential to mitigate HS impacts on growing lambs while improving their antioxidant and immunity status.

## 1. Introduction

Around the world, sheep and lamb production plays a crucial role in the livestock sector; this is especially true in China, which is the largest global sheep producer with 322.3 million head [1]. The primary sheep-producing regions are in the semi-arid northwestern regions where severe heat stress (SHS) routinely occurs. According to the World Meteorological Organization, global warming is projected to continue to increase average surface temperatures by 1.5 °C between 2021 and 2040 [2]. High-temperature environments can lead to heat stress (HS) in ruminants, which results in decreased feed intake and growth performance [3,4], elevated rectal temperature (RT) and respiration rate (RR) [5], and compromised immune system and increased morbidity [3,6]. Heat stress occurs when the heat produced by the animal, combined with ambient environmental heat, exceeds the animal’s ability to dissipate this excess heat through respiration, sweating, and panting, particularly under conditions of high temperature and humidity [7]. Heat stress is a significant environmental stress challenge having adverse effects on growing lamb growth performance, health status, and physiological responses, directly impacting lamb production [8,9] and causing disturbances in metabolic processes [10], endocrine function [11], and immune responses [12].

Feed additives, such as antibiotics, have been used to mitigate negative HS effects [13,14]. But, excessive antibiotic use can lead to antibiotic residues in animal tissues, increase bacterial resistance, and threaten human safety [15]. Many countries and regions, such as China, the United States, and the European Union, have completely banned the use of antibiotics in animal feed [16]. As a result, the development of antibiotic alternatives such as natural feed additives is urgently needed to potentially replace antibiotics. Phytogenic extracts demonstrate great promise as antibiotics alternatives [17]. Essential oils (EOs), rich in compounds like monoterpenes and phenolics, have demonstrated various beneficial properties, including enhanced antioxidant activity, immune response, and antimicrobial effects [18,19,20]. These plant-based compounds have shown promise in improving the health and productivity of animals in hot climates, suggesting they might serve as effective, natural alternatives to antibiotics for mitigating HS and enhancing growth performance and feed efficiency [18,21,22].

Numerous studies have explored the potential of plant EO to replace the benefits previously offered by antibiotics in managing HS [18,22,23,24]. Moreover, combining EO from different plants may offer synergistic effects, enhancing their biological activity. Research indicates that EO blends are more effective at inhibiting pathogens than single EO sources [25,26,27].

The essential oil blend (EOB) used in this study contains Zanthoxylum EO, cinnamon EO, capsicum EO, and carriers such as attapulgite. Zanthoxylum EO, the main component, is known for its anti-inflammatory, antibacterial, antioxidant, and antitumor properties [28,29,30]. It is economically beneficial due to its ease of extraction and widespread availability. Cinnamon EO, derived from cassia cinnamon, is rich in cinnamaldehyde and eugenol, compounds with strong antioxidant and antibacterial properties [31,32,33]. Capsicum EO, extracted from capsicum, contains capsaicin, which is known for its antioxidant and anti-HS effects [34,35,36,37]. Capsaicin is also a known activator of the vanilloid receptor [34] involved in thermoregulation by inducing blood vessel vasodilation to dissipate heat [34]. Vasodilation widens blood vessels to potentially carry more blood to the lungs [38] to increase respiratory evaporation for heat dissipation through panting, etc.

A literature search revealed a paucity of information in the literature evaluating feeding an EOB to growing HS lambs. The hypothesis was that supplementing growing lambs with an EOB would ameliorate HS impacts by enhancing DMI, growth, oxidative, and immune responses. The study objective was to evaluate an EOB fed to growing lambs experiencing mild-to-extreme HS on growth performance, blood metabolites, antioxidant capacity, and physiological responses.

## 2. Materials and Methods

### 2.1. Animal and Receiving Procedures

Prior to experimental initiation, all animal care, handling, and experimental procedures described herein were approved by the Animal Care and Use Committee at Northwest Normal University. (NWNU-CLS-2023-001). In addition, lambs were managed, cared for, and fed following the guidelines published in the 4th edition *“Guide for the Care and Use of Agricultural Animals in Research and Teaching”* published by ADSA-ASAS-PSA [39]. The experiment was conducted at the Shan Hu Husbandry Farm in the Ganzhou District of Zhangye City, Gansu Province, China, during the summer of July and August 2023. Ganzhou District, located in the central region of the Hexi Corridor, has a temperate continental climate, with an average annual sunshine duration of 2932–3085 h, annual solar radiation of 147.99 cal/m^2^, and an annual temperature range from −18 °C to 46 °C. Lambs were housed in a well-ventilated semi-open barn featuring 3 m^2^ per head with a pebble-embedded dirt floor with no additional bedding. Two separate feed troughs were installed with one for grain mix and the second for forage mix consumption, providing approximately 0.75 m linear bunk space per head.

Thirty-two female crossbred (Mongolian × Thin-tailed Han F1) lambs of approximately 3 mo of age (initial BW 18.6 ± 2.43 kg) were selected from a larger group and randomly assigned to 1 of 2 treatments using a completely random design (CRD) [40]. Lambs were grouped with 4 lambs/pen. A 10 d pre-test period (start 1 July 2023) was used to acclimate lambs to the facilities followed by a 45 d experimental period.

Treatments: (1) Control (CON), grain mix without EOB; (2) EOB, EOB supplement added daily to the grain mix at 4 g/kg. The 4 g/kg EOB grain mix concentration was selected based on a preliminary study using 120 crossbred lambs fed the same EOB mix at 0, 4, 6, and 8 g/kg grain mix. The 4 g/kg inclusion rate demonstrated significantly improved DMI compared to control-fed lambs, while the 6 and 8 g/kg inclusion rates significantly reduced DMI, possibly due to reduced palatability. The lamb’s specific EOB g/d intake will depend on grain mix consumption. The EOB was provided by Northwest Normal University’s Institute of New Rural Development (Lanzhou, Gansu, China) and contained 4.34% Zanthoxylum, 1% capsicum, and 1.06% cinnamon EO, with 93.60% attapulgite (magnesium aluminum phyllosilicate clay, i.e., carrier). The key active EO ingredients were 31.62% linalool, 29.94% sabinene, 21.51% limonene, 6.13% capsaicin, 2.56% cinnamaldehyde, and 1.38% eugenol. The EOB was first adsorbed onto attapulgite (the carrier) and then stored in lightproof sealed bags to minimize exposure to light and air, thereby preventing oxidation and preserving its efficacy. Fresh batches of the EOB-premix were prepared weekly to ensure potency.

The formulated basal ration is given in Table 1 consisting of a grain mix and a forage mix. The forage mix was a thoroughly mixed blend of lucerne hay, corn stalks, and wheat straw. The basal ration is formulated to meet or exceed the nutritional guidelines for 20 kg growing lambs gaining 200 g/d [41,42]. Grain and forage were separately fed 10% orts in two distinct troughs. Forage was provided first, followed by grain one hour later, with this sequence applied at both the 06:00 and 18:00 feedings daily. Before each feeding, orts from the previous feeding were collected and weighed to allow for separate calculations of grain and forage intake. During the entire experimental period, the lambs were allowed ad libitum water consumption with water intake being measured every three days. Body weight (BW) was measured at the beginning and end (0 and 45 d) of the experiment.

### 2.2. Weather Data

Ambient temperature and relative humidity data were recorded daily within 3 equal distance pen locations at 08:00, 10:00, 12:00 (noon), 14:00, 16:00, 18:00, and 20:00 using a split anemometer (DLX-1603A, Delixi Co., Ltd., Hangzhou, China). The temperature humidity index (THI) was calculated according to formula published by Hung et al. [43] and Marai et al. [44],

THI = T − (0.31 − (0.31 × RH) × (T − 14.4))

where T = ambient air temperature (°C) and RH = relative humidity (%/100). A daily THI ≤ 22.2 was considered no HS, while 22.2 < THI ≤ 23.3 was considered moderate HS (MHS), and severe HS (SHS) occurred when 23.3 < THI ≤ 25.6 and extreme HS (EXHS) occurred when THI > 25.6. The THI is a widely recognized metric for assessing the level of weather-induced HS experienced by animals [45].

### 2.3. Growth Performance

Lambs were individually weighed on d 0 following a pre-test 10 d acclimation to the facilities and on d 45 prior to the morning feeding (minimal intestinal fill–shrink) using a wireless digital scale (HY-6088, Huaying Scales Co., Ltd., Dongguan, China). Total DMI (grain plus forage) was determined by measuring ad libitum forage and grain DM intakes daily using a digital scale (MT-201, Merlin Scales Co., Ltd., Xiamen, China). Lambs were fed twice/d to achieve 10% orts. Orts were collected at each feeding prior to new feed being supplied. Individual DMI was based total pen DMI divided by 4 lambs. Initial and final individual BW were used to calculated ADG, and total experimental DMI was summed (grain plus forage DMI) to calculated feed efficiency (ADG, g/DMI, g). Residual feed intake was calculated as actual DMI minus predicted DMI based on NRC prediction tables [41]. Lambs were offered ad libitum access to fresh clean water at all times. However, water intake was measured every 3 d by emptying the water tank with thorough cleaning at 8 a.m. Then, 100 kg of clean water was added to the tank. After 24 h, the following day at 8 a.m. the remaining water was measured using a wireless digital scale (HY-6088, Huaying Scales Co., Ltd., Dongguan, China). Then, average daily water intake (ADWI) was calculated per lamb.

### 2.4. Physiological Parameters

Eight lambs were selected per treatment based on the nearest four lambs being above and four lambs below the treatment mean BW for recording rectal temperature (RT), skin temperature (ST) and respiration rate (RR) at 15:00 every 5 d. The RT was collected via an electronic thermometer (MT-118, NISSEI Ivana Jianbao Electronic Technology Co., Guangzhou, China) inserted 3 cm into the rectum and held for 30 s. The ST was recorded using a hand-held thermometer (E31, Shibekang Medical Equipment Co., Shanghai, China) by placing the thermometer’s sensor end between the wool folds with light pressure on the animal’s hide. The RR (breaths/min) data was collected using a stopwatch and a counter when observing the lamb’s abdomen rising and falling in a quiet state [43].

### 2.5. Blood Sampling and Laboratory Analysis

Jugular blood samples were individually collected from each lamb via venipuncture using two 5 mL vacuum tubes using a 0.55 × 20 mm needle (Kangwei Shi Medical Technology Co. Ltd., Shijiazhuang, China). One 5 mL tube contained ethylenediaminetetraacetic acid (EDTA) as an anticoagulant and the other 5 mL tube was a serum separation tube that was anticoagulant-free but contained silicone gel. Blood samples were collected on d 45 prior to the morning feeding. Blood samples were kept on ice and centrifuged (80-2B, Chengyi Instruments Co., Ltd., Suzhou, China) at 1006× *g* for 15 min outdoors (ambient temperature was 28.3 °C). The separated serum was transferred to poly tubes (Corning Incorporated, Corning, NY, USA) and stored frozen at −80 °C (DW-86L, 7281, Haier Co., Ltd., Qingdao, China) for later analysis.

Serum blood samples were analyzed for total superoxide dismutase (T-SOD), total antioxidant capacity (T-AOC), glutathione peroxidase (GSH-Px), adrenocorticotropic (ACTH), growth hormone (GH), insulin like growth factor 1 (IGF-1), gastrin, cholecystokinin (CCK), immunoglobulins A (IgA), M (IgM), and G (IgG), dopamine (DA), interleukin-6 (IL-6), tumor necrosis factor-α (TNF-α), and interferon-γ (IFN-γ) concentrations via enzyme-linked immunosorbent methods. These concentrations were measured using the corresponding ELISA kits (Nanjing Xinfan Biological Technology Co., Ltd., Nanjing, China) following the manufacturer’s recommended methods. Serum concentrations of blood urea nitrogen, glucose, triglycerides, and cholesterol were determined using an automatic biochemical analyzer (Hitachi 7020, Tokyo, Japan) with commercial kits.

Blood samples collected using an anticoagulant were analyzed within 4 h of collection using a fully automated penta-class blood analyzer (BC-5000 Blood Analysis System; Mindray Inc., Sheng Hong Cheng, Beijing, China) following the manufacturer’s protocols. The following methods were used for complete blood count analysis: (1) Electrical impedance principle (Coulter) for white blood cell counts, red blood cell counts, platelet counts, neutrophil, lymphocyte, monocytes, eosinophils, basophils, neutrophil percentage, lymphocyte percentage, monocyte percentage, eosinophil percentage, basophil granulocyte percentage. (2) Hemoglobin assay employs cyanide-free sodium lauryl sulfate (SLS) hemoglobinometry which meets the MCCLS/CLSO H20-A standard for measuring hemoglobin, hematocrit, mean corpuscular volume, mean corpuscular hemoglobin, mean corpuscular hemoglobin concentration, red blood cell distribution width-standard deviation, platelet distribution width, and procalcitonin concentrations. This instrument meets the America Society for Veterinary Clinical Pathology quality assurance guidelines.

### 2.6. Forage and Concentrate Nutrient Analysis

Concentrate and forage (hay, corn stalks, and wheat straw) samples were collected every 15 d during the experiment and stored at room temperature. At the end of the experiment, forage and concentrate samples were analyzed for nutrient composition following standard AOAC International methods [46]: DM (2001.12) was determined using an oven (HB-503-LF, Hanbaek, Republic of Korea); crude protein (CP; 954.01) was analyzed using a Kjeldahl analyzer (K1100F, Hanon, Qingdao, China); neutral detergent fiber (NDF) with amylase (2002.04) and acid detergent fiber (ADF; 973.18) were determined using a fiber analyzer (ANKOM A200, Ankom Inc., Macedon, NY, USA). The metabolizable energy (ME) was calculated according to People’s Republic of China Agricultural Industry Standard (NY/T 816-2021) equations [42]. Ether extract (920.39) was measured using a Soxhlet extraction apparatus (SOX406, Hanon, Qingdao, China). Ash (942.05) was determined using a muffle furnace (SDMF300, Sandegroup, Changsha, China) at 550 °C. Calcium (Ca; 927.02) and phosphorus (P; 964.06).

### 2.7. Statistical Analysis

All data were checked for normality and outliers using the UNIVARIATE procedure of SAS (version 9.4, SAS Institute Inc., Cary, NC, USA) before any statistical analyses were conducted. Box and whisker plots and the Shapiro–Wilk test were used to verify that data were normally distributed (*p* > 0.10). All data were then subjected to least-squares ANOVA for a completely random design (CRD) [40] having 2 treatments via SAS’s PROC MIXED procedure. The statistical model used was

Y_ij_ = µ + T_i_ + Day_j_ + (T_i_ × Day_j_) + Cov + e_ij_

where Y_ij_ = dependent variable, µ = overall mean, T_i_ = treatment, Day_j_ = day, T_i_ × Day_j_ = interaction of treatment by d, Cov = covariate (initial BW), and e_ij_ = residual random error. Pen was tested and found to be nonsignificant at *p* > 0.50. Therefore, the individual lamb was the experimental unit for all parameters except DMI, which was analyzed on a pen basis. Treatment, day, and the interaction of treatment and day were considered fixed effects, while experimental day was considered a repeated measurement in time having a variance component covariance structure. The use of initial BW as a covariate was only significant (*p* < 0.01) for final BW. If parameters were not repeated over days, day was eliminated from the model. When the F-test for treatment was significant (*p* < 0.10), the PDIFF statement separated least-squares means; this is based on the least significant difference method (LSD). Differences among treatments were considered highly significant at *p* < 0.01, significant at *p* < 0.05, and tendencies at 0.05 < *p* ≤ 0.10.

## 3. Results and Discussion

### 3.1. Ration Nutrient Composition

The ration nutrient concentration (Table 2) was calculated from the analyzed concentrate mix and forage nutrient composition based on actual consumption, which was approximately consumed in a 70:30 ratio (Table 3). The EOB addition to the concentrate would have resulted in minimal or no detectable changes in major nutrient concentrations. The calculated ration nutrient concentration would meet or exceed the nutrient guidelines published for growing lambs [41,42].

### 3.2. Weather Data

**Figure 1:**
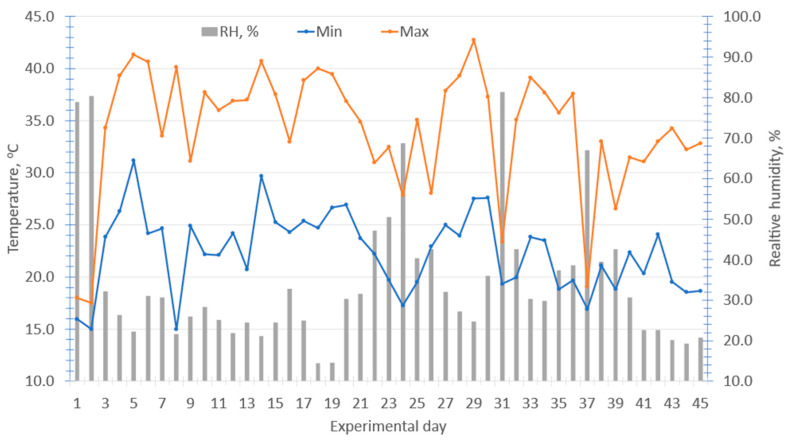
Daily environmental ambient temperature and relative humidity during the 45 d experiment.

**Figure 2:**
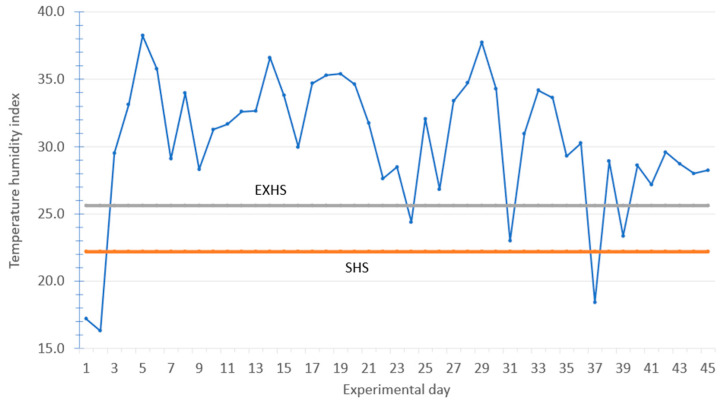
Daily calculated temperature humidity index (THI) during the 45 d experiment. EXHS = extreme heat stress. SHS = severe heat stress.

During the 45 d experiment, the mean daily ambient air temperature was 29.2 ± 4.83 °C ranging from a minimum of 15.0 °C to a maximum of 42.7 °C and the mean daily relative humidity was 34.6 ± 16.8% ranging from a minimum of 14.4% to 81.4% with considerable day-to-day variation during the 45 d experiment (Figure 1). Based on the THI equation and HS classification system proposed by Marai et al. [44], the growing lambs experienced 39 d of EXHS, 2 d of SHS, 1 d of MHS, and only 3 d without experiencing HS out of the 45 d experiment. The average THI was 30.3 ± 5.01, ranging from a low of 16.3 to a maximum of 38.2, indicating that lambs were under EXHS during most of the experiment with considerable variation (Figure 2). The experimental objective of evaluating EOB during HS periods was achieved based on THI values.

### 3.3. Growth Performance

No lambs were identified as outliers. Initial BW values were similar (*p* > 0.10) for lambs fed both treatments and initial BW as a covariate was found to be significant (*p* < 0.01) for adjusting final BW, but was nonsignificant (*p* > 0.10) for all other parameters. Growing HS lambs fed EOB demonstrated greater (*p* < 0.05) final BW and ADG compared with lambs fed CON (Table 3). Feeding EOB resulted in an approximately 50% increase in heat-stressed lamb growth performance.

Lambs fed EOB demonstrated similar (*p* > 0.10) concentrate intake, but greater (*p* < 0.01) forage intake, with greater (*p* < 0.05) total DMI compared with CON-fed lambs. Feeding EOB to HS growing lambs increased forage intake by approximately 20% and total DMI by 7.7% compared with CON-fed HS lambs. These improvements resulted in FE being approximately 47% greater (*p* < 0.01) for HS lambs fed EOB compared to CON-fed HS lambs. A residual feed intake calculation demonstrated similar (*p* > 0.10) residual feed intakes for growing lambs fed both treatments. Water intake and water intake per unit of DMI were similar (*p* > 0.10) for HS lambs fed both treatments.

The primary factors contributing to decreased performance during periods of HS include reduced DMI intake due to peripheral thermal receptors transmitting nerve impulses that inhibit the activity of the hypothalamic appetite center, as well as disruptions in the microbial balance of the digestive system leading to decreased nutrient utilization [44,47]. In this experiment, growing HS lambs supplemented with EOB showed significantly greater growth performance, ADG, and roughage intake compared to CON-fed HS lambs. This improvement can be attributed to the alleviating effects of EOB on HS, which reduces the inhibitory impact on appetite caused by thermal stress. As a result, lambs in the EOB group consumed more DM, leading to better BW ADG. Furthermore, the EOB group demonstrated a significantly higher feed efficiency due to less energy being diverted to maintenance to combat HS impacts.

Much of the research on Zanthoxylum has focused on antimicrobial and preservative effects on food [48,49], with less research on improving ruminant welfare. It has been shown that the addition of 10 mL/kg of *Zanthoxylum bungeanum* to the diet helped to increase rumen pectinase and lipase activities in Small Tail Han lambs [50], suggesting that *Zanthoxylum bungeanum* may promote intestinal health and enhance nutrient digestion and absorption by affecting rumen enzyme activity and the microbiome.

Several studies have shown that feeding capsaicin significantly improved the lactational performance of dairy cows [51,52,53] and improved growth performance in beef cattle [54,55]. In line with this experiment, supplementing 80 mg cinnamaldehyde, eugenol and capsicum oleoresin per kg feed was effective in promoting growth performance and nutrient digestion in growing ewes with stimulatory effects on their immune response and antioxidant status [56]. However, it has been shown that the addition of cinnamon essential oil (1 mL/kg of feed) [57] or capsicum oleoresin (80 mg/kg feed) [58] alone to the diet had no significant effect on DMI, ADG, and FE in sheep. This may be due to EOB supplementation having a growth promotion effect on lambs.

### 3.4. Physiological Indices

**Figure 3:**
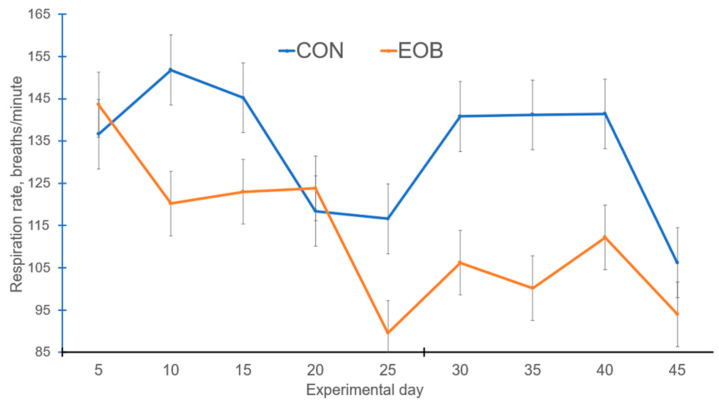
Respiration rate for heat-stressed lambs fed control (CON) or an essential oil blend (EOB). Treatment by day interaction, *p* < 0.05. SEM = 2.76.

**Figure 4:**
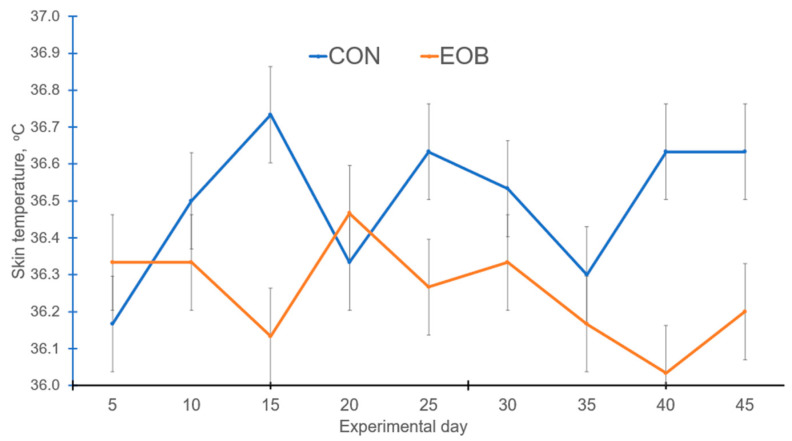
Skin temperature from heat-stressed lambs fed control (CON) or an essential oil blend (EOB). Treatment by day interaction, *p* < 0.05. SEM = 0.04.

**Figure 5:**
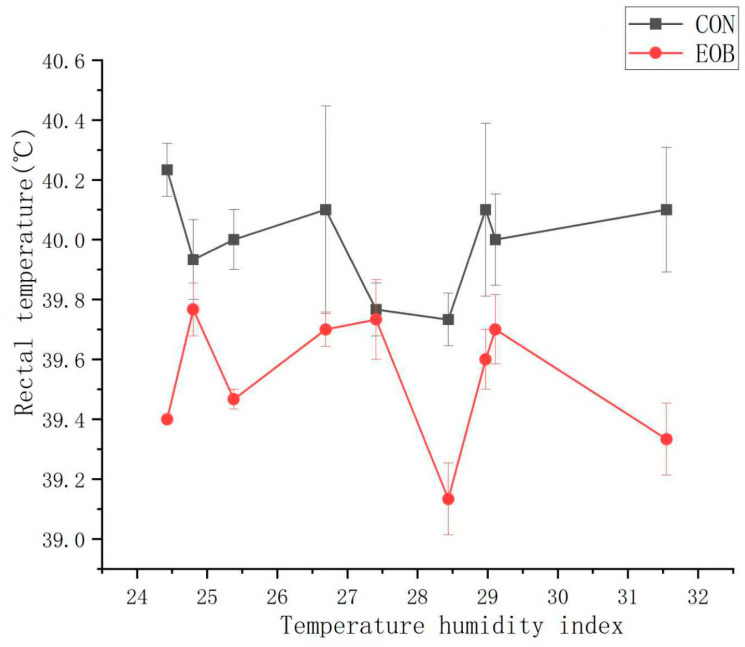
Rectal temperature from heat-stressed lambs fed control (CON) or an essential oil blend (EOB).

**Figure 6:**
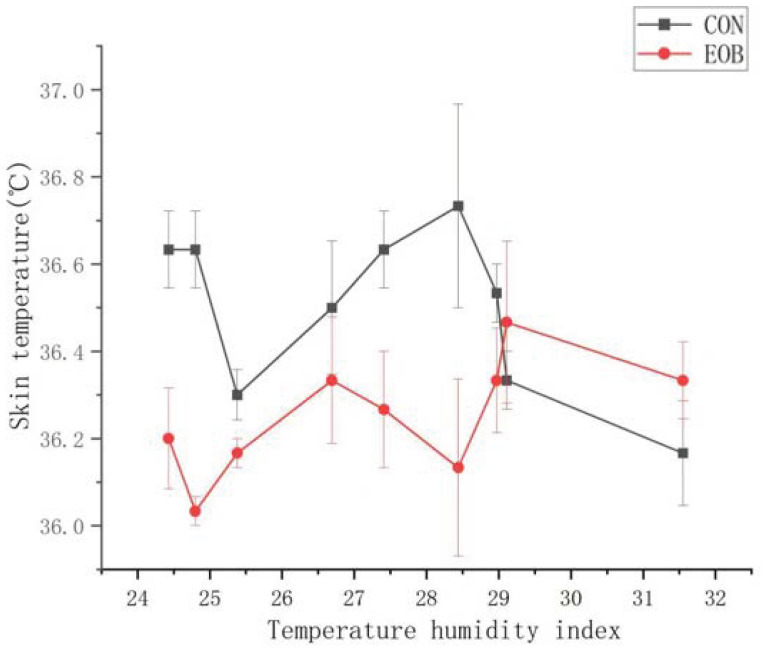
Skin temperature from heat-stressed lambs fed control (CON) or an essential oil blend (EOB).

**Figure 7:**
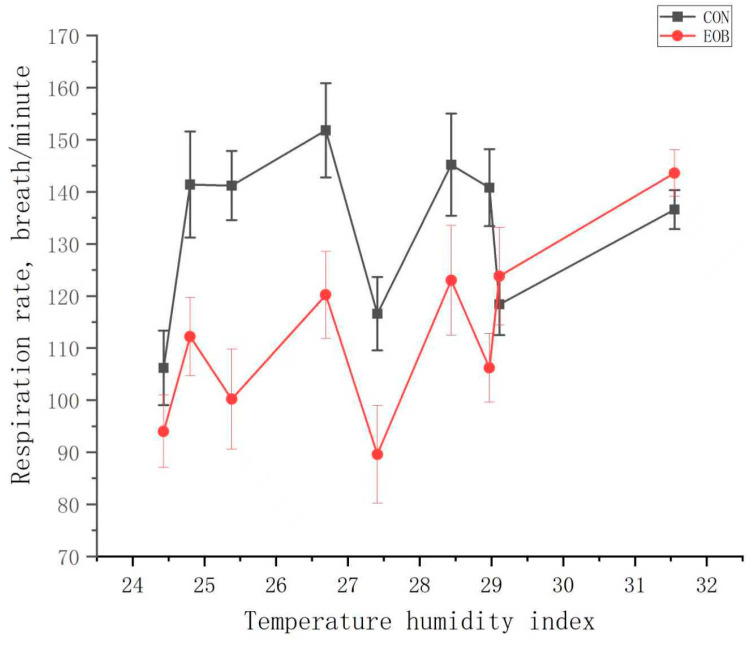
Respiration rates from heat-stressed lambs fed control (CON) or an essential oil blend (EOB).

Lambs fed EOB demonstrated a treatment by day interaction (*p* < 0.05) for RR and ST, but the treatment by day interaction was nonsignificant (*p* > 0.10) for RT (Table 4). Experimental day was significant (*p* < 0.01) for RR, while it was nonsignificant (*p* > 0.10) for RT and ST, but day effects will not be discussed further. Core body temperature is often assessed using the dependable RT, ST, and RR measurements [59]. Lambs fed EOB demonstrated lower (*p* < 0.01) RR compared with CON-fed lambs. During the greatest HS periods, lambs fed EOB consistently demonstrated lower RR compared with CON-fed lambs (Figure 3). The overall experimental RT and ST were lower (*p* < 0.01) for HS lambs fed EOB compared with HS lambs fed CON. During the greatest HS periods, lambs fed EOB consistently demonstrated lower ST compared with CON-fed lambs (Figure 4). An animal’s response to HS is an autonomic response of the autonomic nervous system mediated by catecholamines (epinephrine and norepinephrine), and modulating RR is a mechanism for dissipating excess body heat [60,61]. Respiration is the primary mechanism for heat dissipation as wool limits heat loss; consequently, HS leads to increase RR [62,63]. These data demonstrate that feeding EOB can ameliorate HS impacts in growing HS lambs. The data for RT, ST, and RR by treatment was sorted by THI and plotted to determine if consistent responses occurred over the THI range experienced throughout the experiment. Figure 5, Figure 6 and Figure 7 demonstrate consistent responses in lowering RT, ST, and RR when growing HS lambs were fed EOB compared with lambs fed CON, except for the most extreme THI which was similar for ST (Figure 6) and RR (Figure 7). Baumgard and Rhoads [64] explain that panting is a method of dissipating bodily heat load through evaporative cooling. The reduction in RR would reduce the maintenance energy requirements by reducing the energy expenditure of breathing. These findings indicate that supplementing EOB effectively decreased RR, thus alleviating HS. This reflects the fact that dietary addition of EOB can balance excess calories in sheep by boosting metabolism.

Heat-stressed growing lambs fed an EOB supplement exhibited lower (*p* < 0.01) RT compared to CON-fed lambs. Rectal temperature is often used to assess core body temperature [59], serving as a thermal indicator of changes. A generalized RT increase was reported during HS by increasing external ambient temperatures [65,66]. These data demonstrate that growing HS lambs fed EOB enhanced the regulatory mechanisms to mitigate HS by lowering internal body temperatures.

Feeding EOB to growing HS lambs demonstrated a significant reduction in ST compared with CON-fed HS lambs. During the greatest HS periods, lambs fed EOB consistently demonstrated lower ST compared with CON-fed lambs (Figure 4). De et al. [67] demonstrated that sheep exhibit increased ST in response to HS; thus, ST is a critical physiological indicator for assessing HS. The diminished temperature gradient between the animal and its environment hinders heat dissipation via body surface. This experiment demonstrates that growing HS lambs fed an EOB supplement exhibited lower (*p* < 0.01) ST compared to those in the CON group. Capsicum EO is one of the EOB components that is a known vasodilator for enhancing heat dissipation [68].

### 3.5. Blood Measurements

Of the many serum blood parameters measured in this experiment, only T-SOD and IgM demonstrated tendencies (0.05 < *p* < 0.10) for greater concentrations (Table 5). Heat-stressed lambs fed EOB demonstrated a greater tendency (0.05 < *p* < 0.10) for increased T-SOD concentrations compared with CON-fed HS lambs. Oxidative stress during HS periods results from the lamb generating more reactive oxygen species or reducing antioxidant capacity. Oxidative stress perturbs the delicate equilibrium between oxidative and antioxidant systems preventing tissue damage [69] which can lead to a variety of adverse effects, such as DNA degradation, protein carbonylation, and lipid peroxidation. The main factor contributing to mitochondrial impairment under HS conditions is oxidative stress. At the onset of HS, there is an upsurge in mitochondrial substrate oxidation and electron transport chain activity, leading to heightened production of superoxide [70,71]. Inflammatory responses are increased during HS. Feeding an EOB supplement increased antioxidant (T-SOD) enzymatic activities to reduce inflammation by growing HS lambs. All other serum blood parameters were similar (*p* > 0.10) for HS lambs fed both treatments.

Growing heat-stressed lambs fed EOB demonstrated a tendency for greater (0.05 < *p* < 0.10) IgM concentrations compared with lambs fed CON. Immunoglobulins, as components of humoral immunity, are a class of active molecules that participate in antigen-specific binding and regulation of the immune response. Heat stress leads to impaired immune function. Previous work reported increased serum IgM [56] or IgA, IgG, and IgM concentrations [72,73] by supplementing capsicum to ewes and calves. Liu et al. [25] reported increased blood IgM concentrations when feeding calves an oregano EO product. Our current study revealed that there is a tendency for IgM levels to increase with the inclusion of EOB in the diet, indicating a potential immunomodulatory effect of EOB in growing lambs. Furthermore, a study demonstrated that supplementing 80 mg cinnamaldehyde, eugenol and capsicum oleoresin (CEC) per kg feed was effective in enhancing serum IgG and IgM levels, indicating that CEC might influence the humoral immune responses of ewes [56]. Yang et al. [20] proposed EO as a potential replacement for antibiotic growth promoters. Several studies have reported inconsistent results of enhancing [8,34,74,75] or no response in immune parameters when feeding EO [56,58].

Growing heat-stressed lambs fed EOB demonstrated a lower (*p* < 0.05) eosinophil percentage and a tendency toward lowered (0.05 < *p* < 0.10) MPV compared with lambs fed CON (Table 6). The EOB mechanism of action affecting eosinophils and MPV is unknown. These data are consistent with Rogerio et al. [76], demonstrating various plant extracts and metabolites can inhibit eosinophil recruitment. The EOB used in our study effectively mitigated eosinophils, suggesting a potential shared mechanism of action [76]. Lambs may not be producing as many new platelets due to HS increasing cortisol concentrations. Heat stress impacts the physiological condition of animals, as evidenced by changes in blood biochemical markers that reflect their health and overall physiological status [77].

## 4. Conclusions

Supplementing EOB at 4 g/kg to the grain mix for HS growing lambs in the present experiment improved final BW, BW gain, ADG, forage intake, total DMI, and feed efficiency. Feeding EOB to growing HS lambs reduced RR, RT, and ST, which ameliorated HS impacts on the lamb’s physiological condition. Dietary EOB supplementation can ameliorate HS adverse effects on antioxidant performance, immune status, and physiological responses in sheep, thereby improving growth performance and feed efficiency. Ultimately, this study provides a basis for ration EOB inclusion at 4 g/kg concentrate when feeding growing HS lambs.

## Figures and Tables

**Table 1:** Ingredient composition of formulated basal ration (DM basis, %).

Ingredient Composition	% in Ration	% of Mix
**Forage, mixed**	30.0	
Lucerne		51.0
Corn stalks		25.0
Wheat straw		24.0
**Grain Mix**	70.0	
Corn, ground		63.9
Soybean meal, 48% CP		13.1
Wheat bran		14.9
Sodium bicarbonate		1.00
Calcium biphosphate		0.10
Salt, white		1.00
Soybean oil		1.00
VTM premix ^1,2,3^		5.00

^1^ Content of trace elements in premix: Mn, 603.2 mg/kg; Zn, 904.8 mg/kg; Fe, 822.6 mg/kg; I, 8.2 mg/kg; Se, 8.2 mg/kg; Cr, 24 mg/kg. ^2^ Content of vitamin in premix: vitamin A, 137 IU/g; vitamin D, 24.7 IU/g; vitamin E, 1.65 IU/g. ^3^ The premix was developed by the New Rural Development Research Institute of Northwest Normal University and contracted by Ya Sheng Ben Yuan Biotechnology Co., Ltd. (Lanzhou, China).

**Table 2:** Nutrient composition of individual forages and grain mix and calculated total ration (DM basis).

	Forages			Grain	Calculated
Nutrient	Lucerne	Corn Stalks	Wheat Straw	Mix	Total Ration
DM, %	91.4	92.4	92.7	88.0	89.2
CP, %	15.8	5.84	4.86	15.7	14.2
Ether extract, %	2.54	1.57	1.65	4.51	3.78
ME, MJ/kg ^1^	1.96	1.37	1.56	3.03	2.64
NDF, %	53.6	76.1	68.7	13.2	28.1
ADF, %	36.0	41.3	49.4	5.61	16.1
Ash, %	9.19	8.53	7.66	6.80	7.36
Ca, %	1.72	0.69	0.32	0.76	0.87
P, %	0.26	0.08	0.09	0.64	0.50

^1^ Calculated People’s Republic of China Agricultural Industry Standard (NY/T 816-2021) [42].

**Table 3:** Growth performance, ADG, concentrate, forage, total DMI, and average daily water intake (ADWI) for heat-stressed lambs fed without (CON) or with an essential oil blend (EOB).

	Treatment			*p*< ^1^
Measurement	CON	EOB	SEM	Trt
N	16	16	-----	-----
BW				
Initial, kg	18.6	18.6	0.65	0.98
Final ^2^, kg	23.4 ^b^	25.9 ^a^	0.91	0.02
BW gain, kg	4.83 ^b^	7.29 ^a^	0.91	0.02
ADG, g/d	107.4 ^b^	162.0 ^a^	20.0	0.02
Intake				
Concentrate, g/d	624.2	643.3	14.3	0.29
Forage, g/d	239.2 ^b^	287.0 ^a^	8.35	0.01
Total DMI, g/d	863.4 ^b^	930.2 ^a^	17.9	0.01
Residual feed intake ^3^				
Total DMI, g/d	111.4	83.5	43.5	0.65
Feed efficiency, g/g	0.124 ^b^	0.181 ^a^	0.005	0.01
ADWI, kg/d	4.01	4.11	0.30	0.77
Water/DMI, kg/kg	4.67	4.42	0.44	0.61

^a,b^ Means within a row with unlike superscripts differ (*p* < 0.05). ^1^ Probability of F test for treatment. ^2^ Initial BW was a significant covariate for adjusting final BW. ^3^ Residual feed intake calculated as 3% of final body weight [41].

**Table 4:** Respiration rate (RR), rectal temperature (RT) and skin temperature (ST) of heat-stressed lambs fed without (CON) or with an essential oil blend (EOB).

	Treatment			*p*< ^1^		
Measurement	CON	EOB	SEM	Trt	Day	Trt × Day
N	8	8	-----	-----	-----	-----
RR, breaths/min	133.1 ^a^	112.5 ^b^	2.76	0.01	0.01	0.03
RT, °C	40.0	39.5	0.06	0.01	0.14	0.19
ST, °C	36.5 ^a^	36.3 ^b^	0.04	0.01	0.56	0.03

^a,b^ Means within a row with unlike superscripts differ (*p* < 0.05). ^1^ Probability of F test for treatment.

**Table 5:** Serum measurements by heat-stressed lambs fed without (CON) or with an essential oil blend (EOB).

	Treatment			*p*< ^1^
Measurement	CON	EOB	SEM	Trt
N	8	8	-----	-----
Blood urea nitrogen, mmol/L	6.40	6.38	0.42	0.98
Glucose, mmol/L	2.65	2.65	0.21	0.99
Triglyceride, mmol/L	0.24	0.22	0.02	0.54
Cholesterol, mmol/L	1.50	1.50	0.06	0.94
T-SOD ^2^, U/mL	146.6	163.2	5.96	0.06
T-AOC ^3^, U/mL	4.97	5.10	0.71	0.90
GSH-PX ^4^, U/mL	68.0	71.7	13.3	0.84
ACTH ^5^, pg/mL	88.9	75.6	7.64	0.22
Growth hormone, ng/mL	5.97	7.03	0.67	0.26
IGF-1 ^6^, ng/mL	408.7	488.7	70.0	0.41
Gastrin, pg/mL	131.2	131.7	16.2	0.99
CCK ^7^, ng/mL	3.06	3.27	0.40	0.70
Heat shock protein-70, pg/mL	364.9	370.4	32.0	0.90
Dopamine, pg/mL	355.6	327.6	62.4	0.75
IL-6 ^8^, pg/mL	81.2	79.7	10.3	0.92
TNF-α ^9^, pg/mL	41.5	47.1	5.55	0.46
IFN-γ ^10^, pg/mL	317.4	386.0	29.3	0.11
IgA, μg/mL	74.4	85.5	6.32	0.12
IgG, mg/mL	26.4	27.3	2.86	0.83
IgM, μg/mL	591.1	778.1	72.9	0.08

^1^ Probability of F test for treatment. ^2^ Total superoxide dismutase. ^3^ Total antioxidant capacity. ^4^ Glutathione peroxidase. ^5^ Adrenocorticotropic hormone. ^6^ Insulin like growth factor-1. ^7^ Cholecystokinin. ^8^ Interleukin-6. ^9^ Tumor necrosis factor-α. and ^10^ Interferon-gamma.

**Table 6:** Blood immune parameters by heat-stressed lambs fed without (CON) or with an essential oil blend (EOB).

	Treatment			*p*< ^1^
Measurement	CON	EOB	SEM	Trt
N	8	8	-----	-----
White blood cells, ×10^9^/L	10.7	11.0	0.56	0.72
Neutrophils, ×10^9^/L	5.30	4.87	0.48	0.54
Lymphocytes, ×10^9^/L	2.62	3.33	0.65	0.46
Monocytes, ×10^9^/L	1.69	1.39	0.47	0.66
Eosinophils, ×10^9^/L	0.71	0.57	0.07	0.19
Basophiles, ×10^9^/L	1.11	1.10	0.25	0.99
Neutrophils, %	45.9	46.6	3.80	0.91
Lymphocytes, %	23.2	27.6	5.22	0.56
Monocytes, %	14.8	7.9	3.44	0.18
Eosinophils, %	6.30 ^a^	4.65 ^b^	0.50	0.04
Basophiles, %	9.83	9.99	2.43	0.97
Red blood cells, ×10^12^/L	11.3	11.1	0.28	0.74
Hemoglobin (HGB), g/L	141.3	139.9	3.72	0.80
Hematocrit, %	30.1	30.3	0.69	0.87
Mean corpuscular volume, L	26.8	27.3	0.64	0.64
Mean corpuscular HGB, pg	12.6	12.6	0.28	0.96
Mean corpuscular HGB, g/L	469.0	461.9	6.08	0.43
RBC ^2^ width SD, L	22.9	23.0	0.57	0.97
RBC ^2^ width CV, %	20.2	19.9	0.43	0.72
Platelets, ×10^9^/L	589.3	671.5	65.7	0.40
Mean platelet volume, L	4.26	4.05	0.08	0.08
Procalcitonin, %	0.25	0.30	0.02	0.19

^a,b^ Means within a row with unlike superscripts differ (*p* < 0.05). ^1^ Probability of F test for treatment. ^2^ Red blood cells.

## Data Availability

Data are available on request from the corresponding authors. Due to legal restrictions that are under review for regulatory evaluation. But once these issues are resolved the data can be made available by contacting the corresponding author or will be deposited in a repository.

## Abbreviations

ACTH	Adrenocorticotropic hormone
ADF	Acid detergent fiber
ADWI	Average daily water intake
CEC	Cinnamaldehyde, eugenol, and capsicum oleoresin
CCK	Cholecystokinin
CON	Control treatment
CP	Crude protein
CRD	Completely random design
DA	Dopamine
EDTA	Ethylene diamine tetra acetic acid
EO	Essential oil
EOB	Essential oil blend
EXHS	Extreme heat stress
FE	Feed efficiency
GH	Growth hormone
GSH-Px	Glutathione peroxidase
HS	Heat stress
IgA	Immunoglobulin A
IgG	Immunoglobulin G
IgM	Immunoglobulin M
INF- γ	Interferon gamma
IGF-1	Insulin like growth factor 1
IL-6	Interleukin 6
LSD	Least significant difference
MHS	Moderate heat stress
ME	Metabolizable energy
MPV	Mean platelet volume
NDF	Neutral detergent fiber
RBC	Red blood cells
RH	Relative humidity
RR	Respiration rate
RT	Rectal temperature
SHS	Severe heat stress
ST	Skin temperature
THI	Temperature–humidity index
T-SOD	Total superoxide dismutase
T-AOC	Total antioxidant capacity
TNF-α	Tumor necrosis factor alpha
